# Research and application of a new method for adjusting the vehicle body height

**DOI:** 10.1371/journal.pone.0274108

**Published:** 2022-10-19

**Authors:** Zhihong Li, Jialing Yao, Youlin Xu

**Affiliations:** 1 College of Automobile and Traffic Engineering, Nanjing Forestry University, Nanjing, China; 2 College of Mechanical and Electronic Engineering, Nanjing Forestry University, Nanjing, China; Southwest Jiaotong University, CHINA

## Abstract

The use of active suspension for vehicle height adjustment has problems of high cost, high energy consumption, slow response, and complex structure. This paper proposes a new method for adjusting the vehicle body using the damping asymmetric characteristic of semi-active suspensions, which is based on the idea that the dampers with damping asymmetric characteristics will cause a change in the mean position of the vehicle body vibration. To verify the feasibility of this method, a single-wheel vehicle model containing asymmetric damping is established. The system’s responses under the sinusoidal and random roads excitation are obtained by the fourth-order Runge-Kutta method, the influences of key parameters on the vehicle body’s shifting height are analyzed, and the mechanism of vehicle body’s shift is explained from the perspective of energy conservation. Then a vehicle body height controller based on third-order linear active disturbance rejection control (LADRC) is designed. Simulation results show that the proposed method for controlling the vehicle height with asymmetric damping can quickly adjust the vehicle to the expected height whether under the sinusoidal road or random road. In addition, no additional hardware and energy consumption are required, providing a new idea for vehicle height control.

## 1. Introduction

Vehicle height adjustment can improve vehicle handling stability and passing ability on bad road [1, 2]. The vehicle height control is mainly based on active suspension, and the adopted actuators are mainly air suspension and hydraulic suspension [3, 4], which have the problems such as large energy consumption, high cost, complex structure, and slow response [5–7]. In addition, the vehicle shifting system based on active suspension requires the installation of additional vehicle shifting devices, and the structure is very complex, for example, the vehicle height adjustment system based on air suspension inflates and deflates the air on the spring through the air pump to change the vehicle body height, and the system requires additional components such as an air pump, an air storage tank, and an air filling and deflating line [8, 9]. The vehicle height adjustment based on hydro-pneumatic suspension uses a hydraulic oil pump to charge or discharge oil on the hydraulic cylinder, and the vehicle height is adjusted by the movement of the hydraulic piston, the system requires the installation of a hydraulic oil pump, oil tank, hydraulic piping and other components [10, 11].

Nevertheless, semi-active suspensions achieve improvements in suspension characteristics by adjusting suspension stiffness or damping, only requiring little energy consumption, which have the advantages of simple structure and low cost [12–14]. If the semi-active suspension can be used to adjust the vehicle height, the vehicle height control can be realized at a lower cost and energy consumption, without installing additional vehicle body shifting devices, which has great practical application value. However, so far, there are no reports about using semi-active suspensions to shift the vehicle body.

In this paper, a novel idea of adjusting the vehicle body height based on a semi-active actuator is proposed, and its origin and basis are as follows. Most passive suspension dampers have unequal damping forces in the compression travel and extension travel, the dampers have damping asymmetric characteristics to achieve a better compromise between vehicle ride comfort and handling stability [15]. Warner [16] tested the damper with such damping asymmetric characteristic and found that the mean position of the vehicle body showed a considerable downward movement, and the magnitude of the movement displacement was related to damping coefficients in the compression travel and that in the extension travel. He defined the phenomenon that the asymmetric characteristics of semi-active suspensions cause a change in the mean position of the vehicle body as ‘shift phenomenon’. Balike [17] established a quarter vehicle dynamics model with a double-wishbone suspension and further proved the existence of the phenomenon that the mean position of the vehicle body vibration moves downward under harmonic excitation. C. Rajalingham [18] solved the exact solution of a quarter vehicle system by the Laplace transform method, it was found that the asymmetric damping of the damper leads to a change in the compression volume of the suspension spring, resulting in a shift in the mean position of the vehicle body, but this asymmetric damping does not cause a change in the mean position of the unsprung mass. M. Silveira [19] established a single-degree-of-freedom oscillator with damping asymmetric characteristic and solved the accurate analytical solutions of all possible combinations of the system under underdamped, critically damped, overdamped, and undamped conditions, arguing that the asymmetric damping caused the difference in the time spent by the isolated body in the compression and extension travels, resulting in the change in the mean position of the vibration of the isolated body. Fernandes [20] presented a vehicle model with asymmetric damping, it was found that the asymmetric damping causes a change in the mean position of the vehicle body, however, it does not induce significant changes in the displacement amplitude of the vehicle body. Summarize this phenomenon: the suspension with damping asymmetric characteristic will cause a change in the mean position of the vehicle body vibration, and the magnitude of the displacement is related to the damping coefficient in the extension travel and compression travel [16–20]. However, existing studies take passive suspension dampers as the research object, and are limited to studying the effects of the change in the vehicle body’s mean position on vehicle driving performances and do not involve how this phenomenon can be applied in engineering.

This paper draws on the phenomenon that the asymmetric damping causes a change in the mean position of the vehicle body, and innovatively proposes a method for shifting the vehicle body by utilizing the damping asymmetric characteristic of the semi-active suspension, the semi-active suspension damping-adjustable damper is used to replace the passive suspension damper, and the damping coefficients in the extension travel and the compression travel are adjusted to control the vehicle body to the expected height. In addition, this method can also be applied to fields such as vehicle attitude control and shifting control of other isolated bodies, for example, the vehicle rolling control and active tilt control [21, 22]. To verify the feasibility of this method and to implement vehicle height control, a single-wheeled vehicle model with damping asymmetric characteristic is established. The Runge-Kutta method is widely used in engineering and has high accuracy [23, 24], thus the fourth-order Runge-Kutta method is used to analyze the system numerically, the feasibility of shifting the vehicle height with asymmetric damping is verified, the influences of relevant parameters on the vehicle body’s shifting height are analyzed, and the intrinsic mechanism of the vehicle body’s shift is investigated from the perspective of energy conservation. Further, a three-order LADRC is designed for the study of vehicle height control.

The following parts of the paper are structured as follows. The first part describes the basic idea of shifting the vehicle body’s height by utilizing the damping asymmetric characteristic of the semi-active suspension. The second part solves the system’s responses under sinusoidal roads and random roads by using the fourth-order Runge-Kutta method, the feasibility of the proposed method for shifting the vehicle body is preliminary verified, the influences of key parameters on the vehicle body’s shifting height are analyzed, and the internal mechanism of vehicle body’s shift is investigated from the perspective of energy conservation. In the third part, the third-order LADRC is designed to control the vehicle body height. The fourth part draws some conclusions.

## 2. Basic idea of shifting the vehicle body

The ratio of the damper’s damping coefficient in the extension travel to the damping coefficient in the compression travel is defined as the damping asymmetric ratio *β*

$$\beta =\frac{c^{\left(+\right)}}{c^{\left(-\right)}}$$ (1)

where *c*^(+)^ is the damping coefficient in the extension travel, *c*^(-)^ is the damping coefficient in the compression travel.

**Fig 1:**
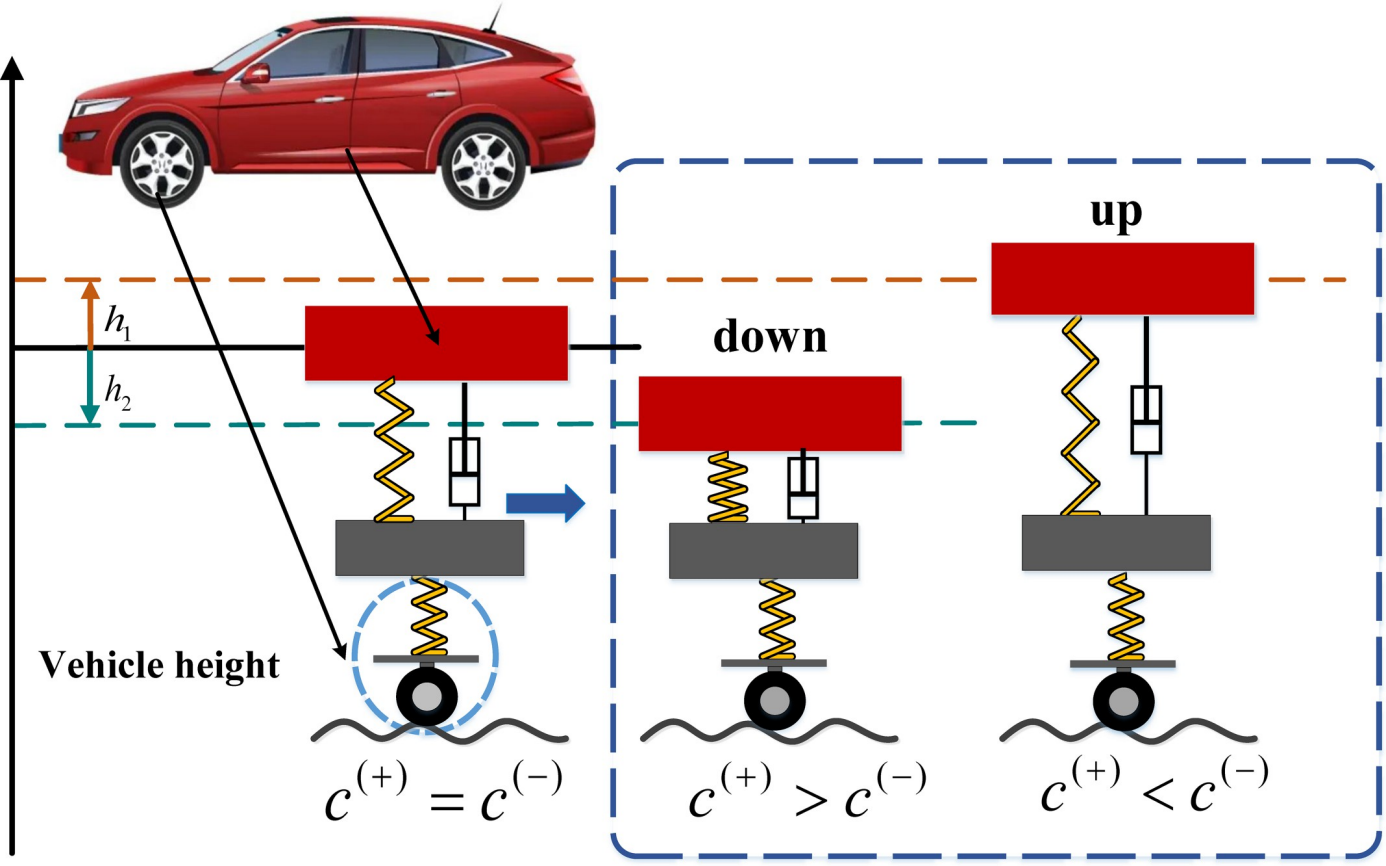
Diagram of the height change in the vehicle body.

A passive suspension damper with damping asymmetric characteristic will cause a change in the mean position of the vehicle body vibration, as shown in Fig 1, when *c*^(+)^ > *c*^(-)^, the mean position of the vehicle body vibration drops by *h*_2_; when *c*^(+)^ < *c*^(-)^, the mean position of the vehicle body vibration raises by *h*_1_; when *c*^(+)^ = *c*^(-)^, there is no change in the mean position of the vehicle body vibration, the magnitudes of *h*_1_ and *h*_2_ are related to the damping coefficients in the extension travel and compression travel [16–20].

**Fig 2:**
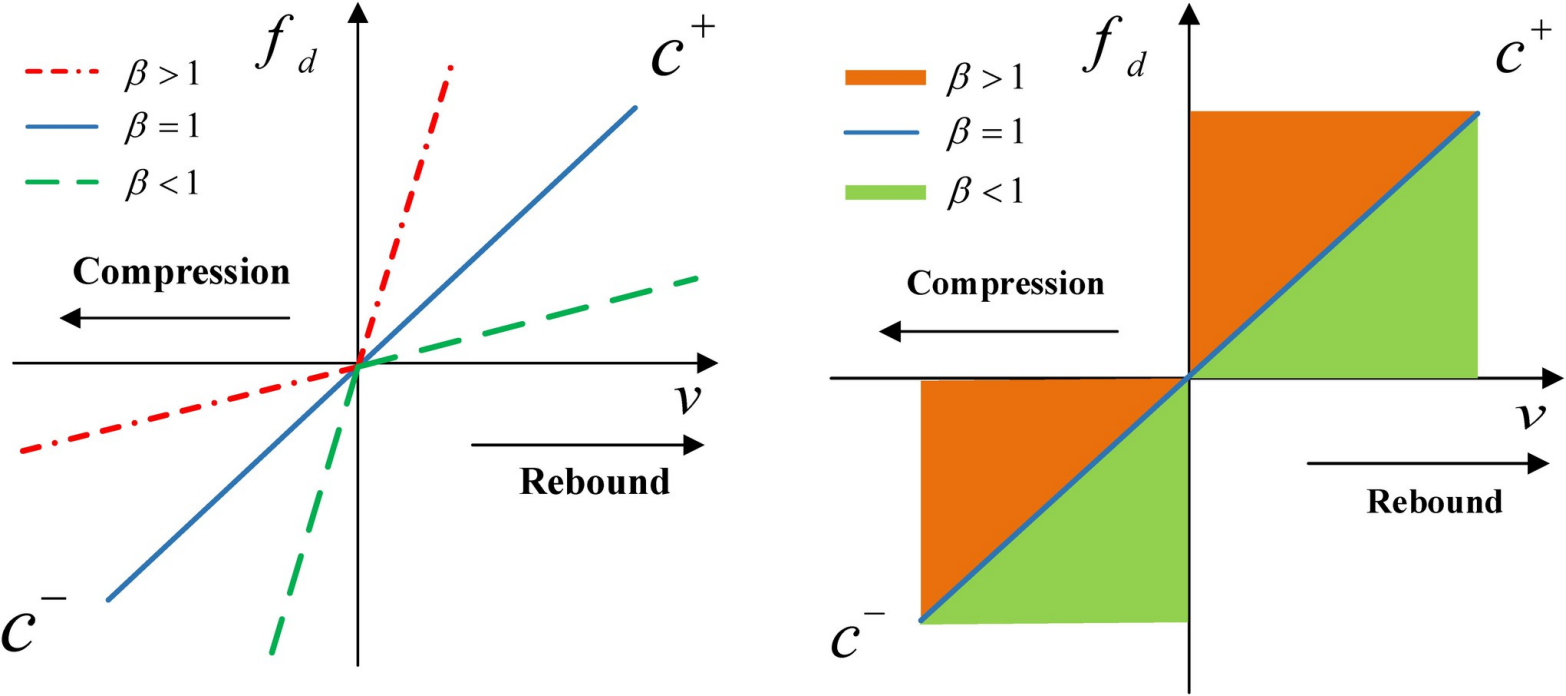
Damping force-velocity characteristic curves. (A) Passive suspension damper; (B) Damping-adjustable damper.

The damping coefficient of the passive suspension damper is fixed, while the damping coefficient of the damping-adjustable damper is continuously adjustable within a certain range. Fig 2 shows the force-velocity characteristic curves of the passive suspension dampers and the damping-adjustable dampers, *f*_*d*_ is the damping force of the damper, *v* is the relative velocity of the vehicle body and wheel, the damping coefficient of the damper *c* = *f*_*d*_/*v*. *v*>0 indicates that the damper is in the extension travel, the damping coefficient is *c*^(+)^, *v*<0 indicates that the damper is in the compression travel, and the damping coefficient is *c*^(−)^. Fig 2A shows the force-velocity characteristic curves of the three passive suspension dampers, the parameters of the passive suspension damper are determined after design, and the value of the damping asymmetric ratio *β* is fixed, therefore, the displacement of the change in the vehicle body’s mean position caused by damping asymmetric characteristic cannot be changed. Fig 2B shows the force-velocity characteristic of the damping-adjustable damper. According to Eq (1), the value of *β* is determined by *c*^(+)^ and *c*^(−)^, the damping-adjustable damper can change the damping coefficients in the extension travel and compression travel, so the damping asymmetric ratio *β* is continuously adjustable within a certain range. If the damping-adjustable damper is used to replace the passive suspension damper, the mean position of the vehicle body can be changed by changing the damping asymmetric characteristic of the suspension, and then the vehicle body height can be adjusted.

### 3. Numerical analysis and discussion

**Fig 3:**
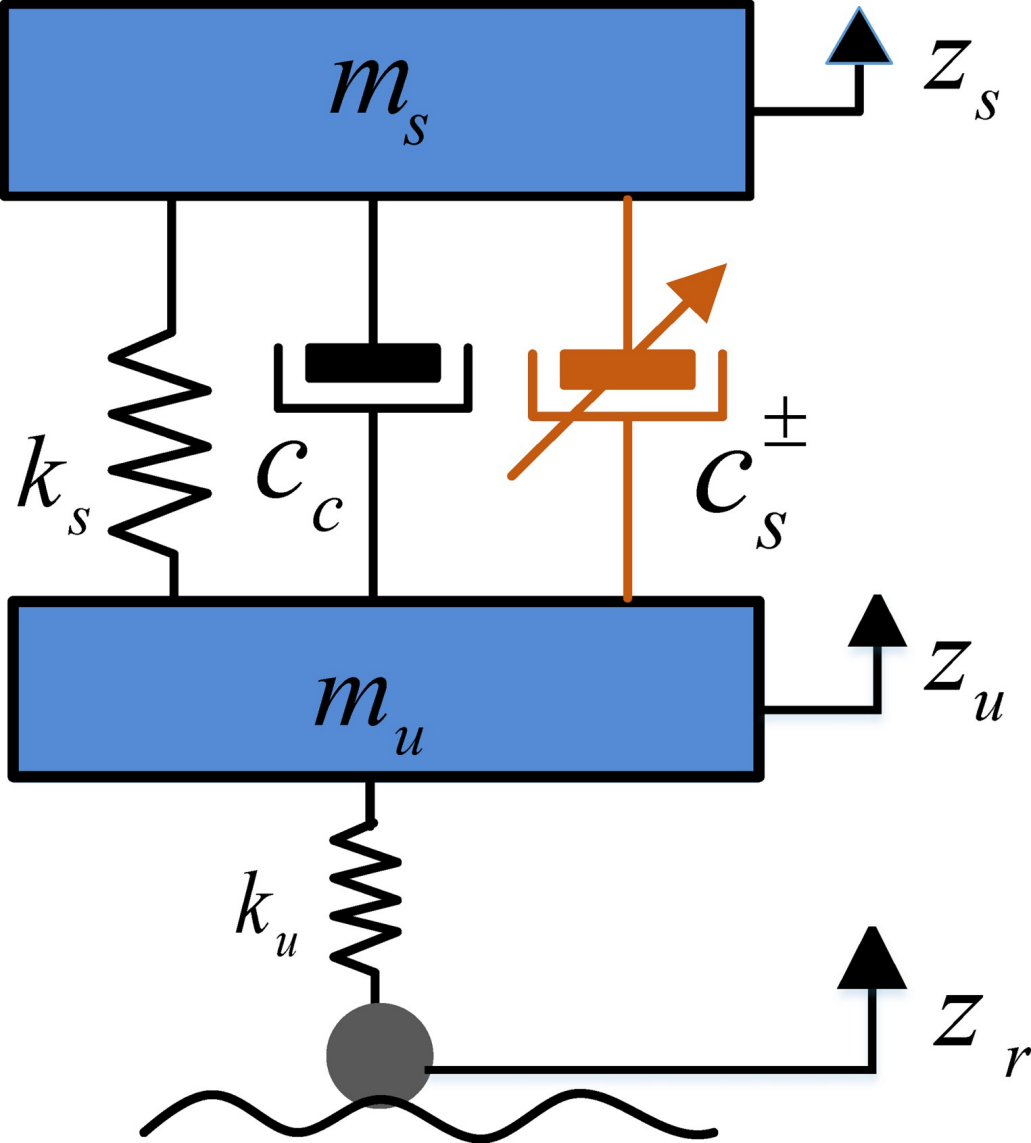
2-degree-of-freedom vehicle vibration model.

A 2-degree-of-freedom vehicle vibration model containing asymmetric damping is established, as shown in Fig 3.

The dynamics equations of the 2-degree-of-freedom vehicle model shown in Fig 3 are established according to Newton’s second law

$$\left\{ \begin{array}{l} m_{s}{\ddot{z}}_{s}+k_{s}(z_{s}-z_{u})+c_{c}({\dot{z}}_{s}-{\dot{z}}_{u})+c_{s}^{\pm }({\dot{z}}_{s}-{\dot{z}}_{u})=0\\ m_{u}{\ddot{z}}_{u}+k_{s}(z_{u}-z_{s})+c_{c}({\dot{z}}_{u}-{\dot{z}}_{s})+k_{u}(z_{u}-z_{r})-c_{s}^{\pm }({\dot{z}}_{s}-{\dot{z}}_{u})=0 \end{array}\right.$$ (2)

where *m*_*s*_ is the vehicle body mass; *m*_*u*_ is the wheel mass; *k*_*s*_ and *k*_*u*_ are the suspension stiffness and tire stiffness; *z*_*s*_ is the displacement of the vehicle body; *z*_*u*_ is the displacement of the wheel; *z*_*r*_ is the road input; *c*_*c*_ is the inherent damping coefficient of suspension; $c_{s}^{\pm }$ is the asymmetric damping coefficient.

$$c_{s}{}^{\pm }=\{\begin{array}{c} c_{s}^{\left(+\right)}\;if\;{\dot{z}}_{s}(t)-{\dot{z}}_{u}(t)\geq 0\\ c_{s}^{\left(‐\right)}\;if\;{\dot{z}}_{s}(t)-{\dot{z}}_{u}(t)<0 \end{array}$$ (3)

where $c_{s}^{\left(+\right)}$ is the damping coefficient in the extension travel; $c_{s}^{\left(‐\right)}$ is the damping coefficient in the compression travel.

For the convenience of numerical calculation, let *x*_1_ = *z*_*s*_, $x_{2}={\dot{z}}_{s}$, *x*_3_ = *z*_*u*_, $x_{4}={\dot{z}}_{u}$, Eq (2) can be written as

$$\left\{ \begin{array}{l} {\dot{x}}_{1}=x_{2}\\ {\dot{x}}_{2}=[k_{s}^{}(x_{3}-x_{1})+c_{c}(x_{4}-x_{2})+c_{s}^{\pm }(x_{4}-x_{2})]/m_{s}\\ {\dot{x}}_{3}=x_{4}\\ {\dot{x}}_{4}=[k_{s}(x_{1}-x_{3})+c_{c}(x_{2}-x_{4})+c_{s}^{\pm }(x_{2}-x_{4})-k_{u}(x_{3}-z_{r})]/m_{u} \end{array}\right.$$ (4)

Eq (2) is solved numerically by the fourth-order Runge-Kutta method to preliminarily verify the feasibility of shifting the vehicle body by utilizing the damping asymmetric characteristic, the influences of key parameters on the vehicle body’s shifting height are analyzed, and the internal mechanism of vehicle body’s shift is explored from the perspective of energy conservation. Select the vehicle parameters [25]: *m*_*s*_ = 410 kg, *m*_*u*_ = 39 kg, *k*_*s*_ = 20000 N/m, *k*_*u*_ = 183000 N/m, *c*_*c*_ = 1200 N⋅s/m.

### 3.1. Solution analysis under sinusoidal road excitation

The sinusoidal road waveform can be expressed as

$$z_{r}=A\sin(wt)$$ (5)

where *A* is the road excitation amplitude, *w* is the road excitation frequency, take *A* = 3 cm, *w* = 20 rad/s.

#### 3.1.1. Feasibility verification

**Fig 4:**
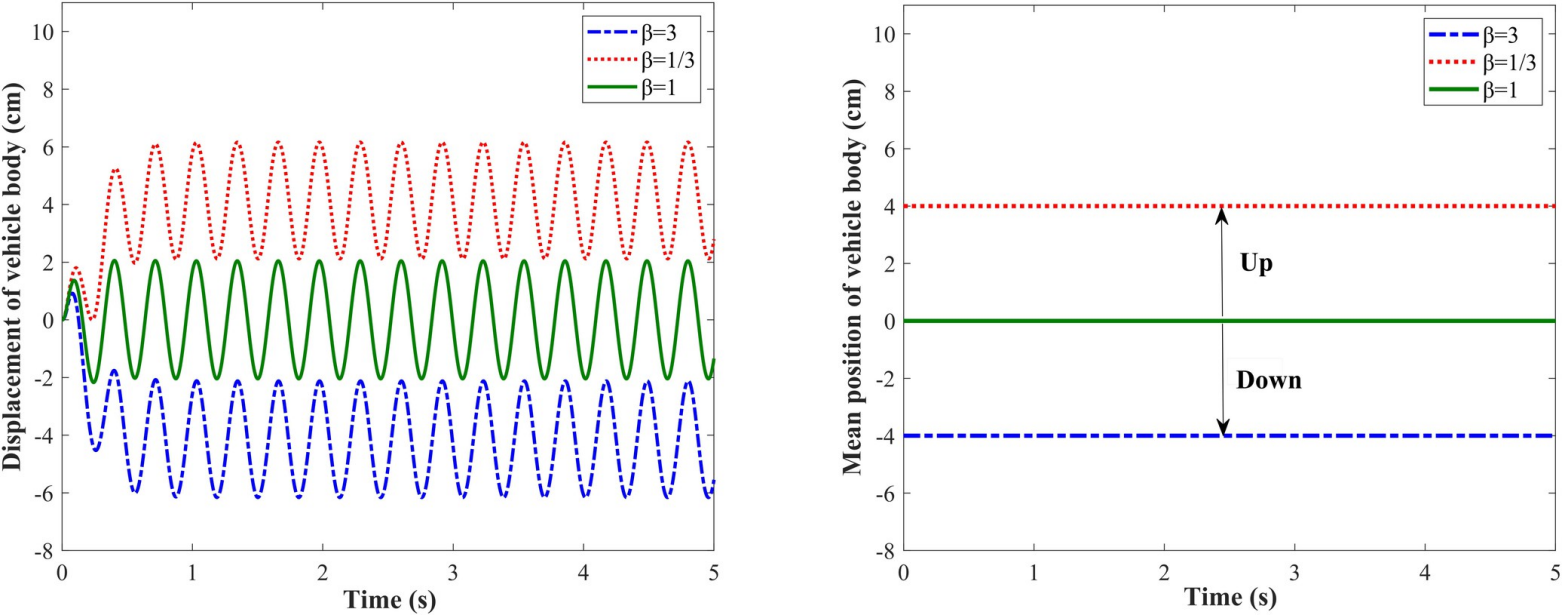
Vehicle body responses under sinusoidal road excitation. (A) Displacement of the vehicle body; (B) Mean position of the vehicle body.

Three different damping asymmetric ratios are taken for simulation. (1) $c_{s}^{\left(+\right)}$ = 6000 N⋅s/m, $c_{s}^{\left(‐\right)}$ = 2000 N⋅s/m, according to Eq (1), *β* = 3; $c_{s}^{\left(+\right)}$ = 2000 N⋅s/m, $c_{s}^{\left(‐\right)}$ = 6000 N⋅s/m, *β* = 1/3; $c_{s}^{\left(‐\right)}$ = 2000 N⋅s/m, $c_{s}^{\left(+\right)}$ = 2000 N⋅s/m, *β* = 1. Fig 4A shows the displacement response of the vehicle body under the sinusoidal road excitation, the proposed method for shifting the vehicle body dynamically adjusts the vehicle body height by using a damping-adjustable damper during the driving process of the vehicle, the vehicle body oscillates under road excitation, and the mean position of the oscillation is the current vehicle body height, as shown in Fig 4B. Set the upward direction of the vehicle body to be positive, as shown in Fig 4, *β* = 1 indicates $c_{s}^{\left(+\right)}$ = $c_{s}^{\left(-\right)}$, the damper does not have damping asymmetric characteristic, at this time, the mean position of the vehicle body vibration is 0 cm; *β* = 3 indicates $c_{s}^{\left(+\right)}$ > $c_{s}^{\left(-\right)}$, the damping coefficient in the extension travel is greater than that in the compression travel, and the mean position of vehicle body vibration drops to -4 cm. *β* = 1/3 indicates $c_{s}^{\left(+\right)}$ < $c_{s}^{\left(-\right)}$, the damping coefficient in the extension travel is smaller than that in the compression travel, and the mean position of vehicle body vibration raises to 4 cm. It can be seen that the proposed method for shifting the vehicle body by utilizing the damping asymmetric characteristic of semi-active suspension is feasible under the sinusoidal road excitation.

#### 3.1.2. Influence laws of parameters

**Fig 5:**
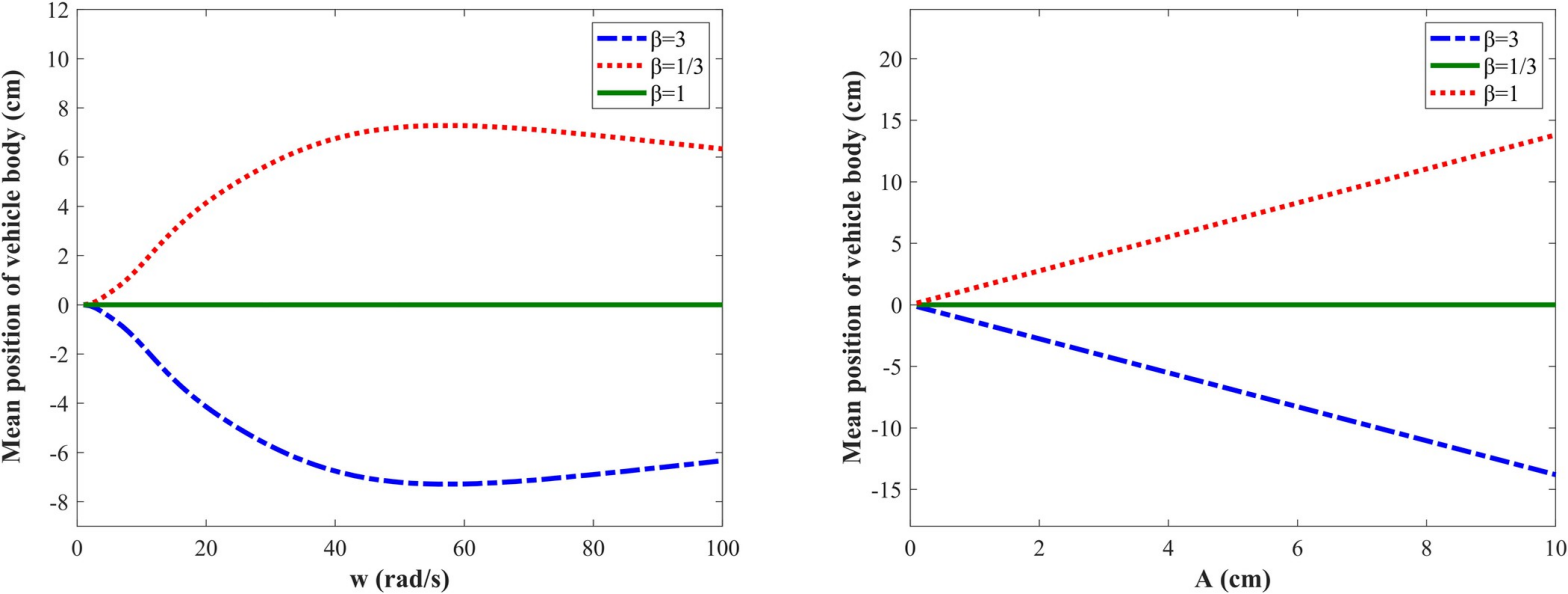
Mean position response of vehicle body under different road excitation. (A) Varying road frequencies; (B) Varying road amplitudes.

Firstly, the influence laws of road excitation amplitude, frequency on the vehicle body’s shifting height are studied. Fig 5A shows the variation curves of the vehicle body’s mean position when the excitation amplitude *A* = 3 cm and excitation frequency *w* changes from 1 to 100 rad/s. When *β* = 3, the mean position of the vehicle body drops; when *β* = 1/3, the mean position of the vehicle body raises; when *β* = 1, the mean position of the vehicle body remains unchanged. In the range of 0 ~ 40 rad/s, the vehicle body’s shifting height increases rapidly with the increase of the road frequency, and the increased rate slows down when the frequency is in the range of 40 ~ 60 rad/s; when the frequency reaches 60 rad/s, the vehicle body’s shifting height reaches the maximum value of 7 cm and then starts to decrease. It is calculated that the wheel’s inherent frequency is 11 Hz, so the vehicle body’s shifting height reaches the maximum around the wheel’s inherent frequency. Fig 5B shows the variation curve of the vehicle body’s mean position with the excitation amplitude for the frequency *w* = 20 rad/s. The shifting height increases with the increase of the road excitation amplitude when the road excitation frequency is constant.

**Fig 6:**
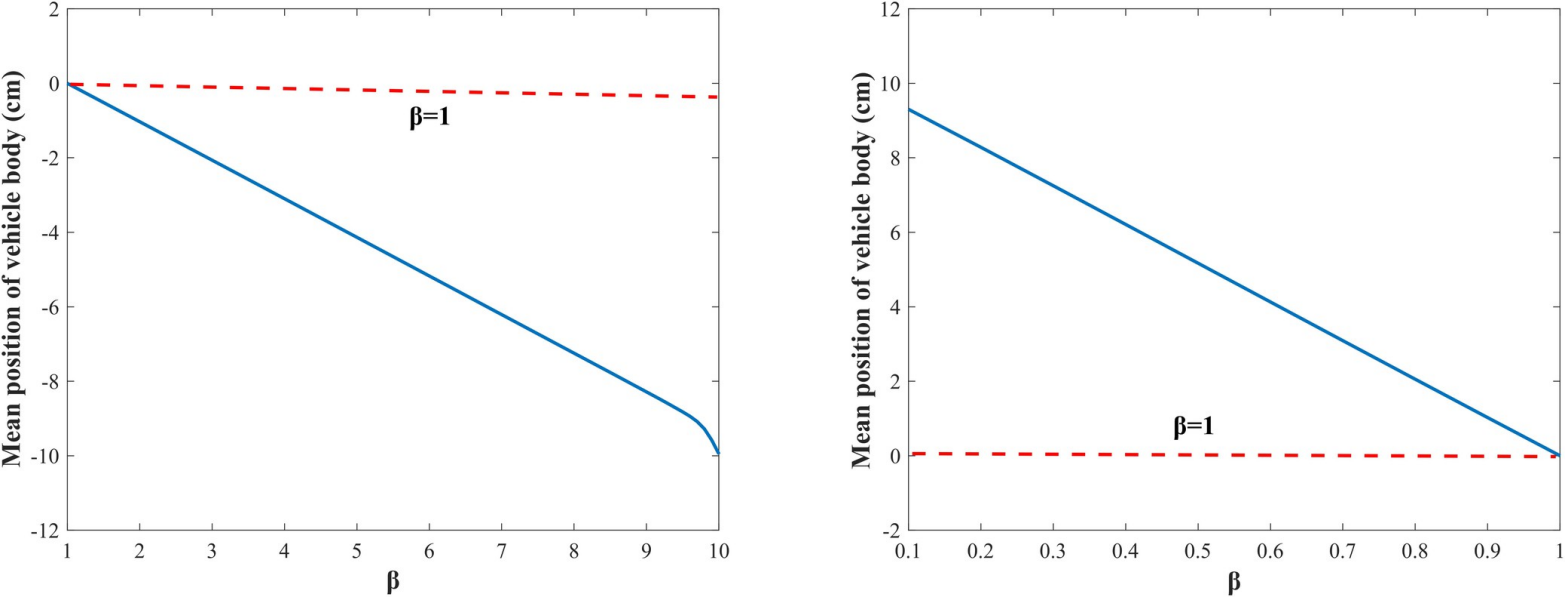
Vehicle body’s mean position at different *β*. (A) Vehicle body drops; (B) Vehicle body raises.

Then the influences of the damping asymmetry ratio on the vehicle body’s shifting height are investigated. Fig 6 shows the variation curves of the vehicle body’s mean position with *β* when the excitation amplitude *A* = 3 cm and the frequency *w* = 20 rad/s. Take $c_{s}^{\left(‐\right)}$ = 1000 N⋅s/m, and the range of *β* is set to [1 10]. At this time, $c_{s}^{\left(+\right)}$ > $c_{s}^{\left(‐\right)}$ according to Eq (1). As shown in Fig 6A, the mean position of the vehicle body drops, and the dropping height increases with the increase of *β*. Take *c*^−^ = 10000 N⋅s/m and the range of *β* is set to (0 1]. At this time, $c_{s}^{\left(+\right)}$ < $c_{s}^{\left(‐\right)}$. As shown in Fig 6B, the mean position of the vehicle body raises and the raising height decreases with the increase of *β*. This is because, when *β*∈[1 10], the larger the value of *β* is, the greater the difference between $c_{s}^{\left(‐\right)}$ and $c_{s}^{\left(+\right)}$ is, the more pronounced the damping asymmetric characteristic is, and the greater the dropping height is; when *β*∈(0 1], the smaller the *β* is, the more pronounced the damping asymmetric characteristic is, and the greater the raising height of the vehicle body is. It can be seen from Fig 6 that the vehicle body’s shifting height is greatly affected by the damping asymmetric ratio, the damping-adjustable damper can control the damping asymmetric ratio by controlling the damping coefficients in the extension and compression travels, so we can adopt the damping-adjustable damper to adjust the vehicle body’s shifting height.

#### 3.1.3. Exploration of the mechanism of vehicle body’s shift

According to the Fourier transform theorem, any complex signal can be decomposed into a superposition of several single-frequency signals [26], therefore, the sinusoidal signal is used to study the internal mechanism of the phenomenon that the asymmetric damping induces a change in the mean position of the vehicle body.

**Fig 7:**
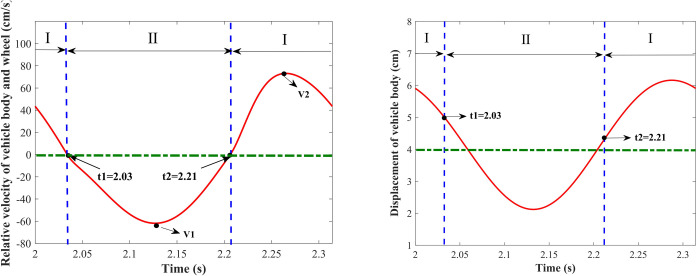
Curves of the relative velocity of vehicle body-wheel and vehicle body displacement in one cycle. (A) Relative velocity; (B) Displacement of the vehicle body.

**Fig 8:**
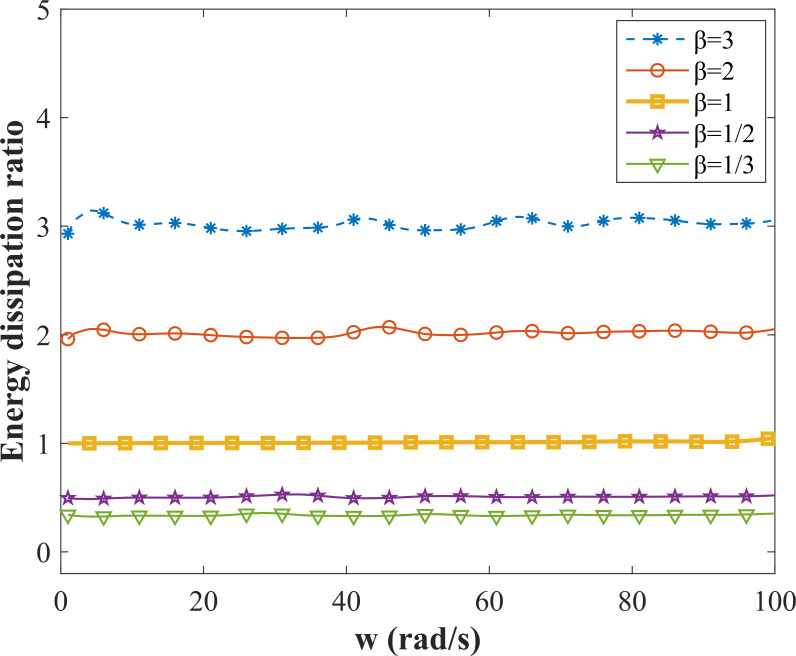
Ratio of energy consumption in extension travel and compression travel.

The ratio of the energy consumed by the damper in the extension travel and compression travel in one cycle is expressed as

$$\mu =\frac{\int_{\tau }^{t_{1}}c_{s}^{\left(+\right)}{\left({\dot{z}}_{s}-{\dot{z}}_{u}\right)}^{2}dt+\int_{t_{2}}^{\tau +2\pi /w}c_{s}^{\left(+\right)}{\left({\dot{z}}_{s}-{\dot{z}}_{u}\right)}^{2}dt}{\int_{t_{1}}^{t_{2}}c_{s}^{\left(-\right)}{\left({\dot{z}}_{s}-{\dot{z}}_{u}\right)}^{2}dt}$$ (6)

where ${\dot{z}}_{s}-{\dot{z}}_{u}$ represents the relative velocity of the vehicle body and wheel, when ${\dot{z}}_{s}-{\dot{z}}_{u}\geq 0$, the damper is in the extension travel; when ${\dot{z}}_{s}-{\dot{z}}_{u}$ < 0, the damper is in the compression travel. *t*_1_ and *t*_2_ are the corresponding times when ${\dot{z}}_{s}-{\dot{z}}_{u}$ = 0, *τ* represents a moment after the calculation results are stable. Fig 7 shows the variation curves of the relative velocity of the vehicle body-wheel and the vehicle body displacement with time respectively in one cycle, starting from 2 s (*τ* = 2) after the calculation results are stable, where $c_{s}^{\left(+\right)}$ = 2000 N⋅s/m, $c_{s}^{\left(-\right)}$ = 6000 N⋅s/m, *A* = 3 cm, *w* = 20. When *t*∈[2.03 2.21], the relative velocity of the vehicle body and wheel is less than 0, and the damper is during the compression travel; when *t*∈[2 2.03]∪*t*∈[2.21 2.314], the relative velocity of the vehicle body and wheel is greater than 0, and the damper is in the extension travel. *μ* ≈ 0.33 can be calculated according to Eq (6), that is, in a cycle, the energy consumed by the damper in the expansion travel is about 1/3 of the energy consumed in the compression travel. As shown in Fig 7B, the mean position of the vehicle body vibration raises to 4 cm at this time. Fig 8 shows the ratio of the energy consumed in the extension and compression travels under the different values of *β* with a fixed excitation amplitude *A* = 3 cm and excitation frequencies taken from 0 to 100 rad/s. *μ* > 1 when *β* > 1, that is, the energy consumed in the extension travel is greater than that in the compression travel. Combined with Fig 6, it can be seen that the mean position of the vehicle body drops, and the larger the value of *β* is, the larger the value of *μ* is, the greater the dropping height of the vehicle body is. *μ* = 1 when *β* = 1, the energy consumed in the extension travel is equal to that in the compression travel, and the mean position of the vehicle body remains unchanged. *μ* < 1 when *β* < 1, that is, the energy consumed in the extension travel is less than that in the compression travel, the mean position of the vehicle body raises, and the smaller the value of *β* is, the smaller the value of *μ* is, and the greater the raising height of the vehicle body is.

With a sinusoidal input with excitation amplitude *A* = 3 cm and excitation frequency *w* = 20 rad/s, and with different damping coefficients, the maximum velocities of the vehicle body in the extension travel and compression travel in one cycle after stabilization are shown in Table 1, where *v*_*com*_ indicates the maximum velocity of the vehicle body in the compression travel; *v*_*ext*_ indicates the maximum velocity of the vehicle body in the extension travel. When $c_{s}^{\left(+\right)}$ > $c_{s}^{\left(-\right)}$, the mean position of the vehicle body drops, at this time, *v*_*ext*_ < *v*_*com*_. When $c_{s}^{\left(+\right)}$ = $c_{s}^{\left(-\right)}$, the mean position of the vehicle body remains unchanged, *v*_*ext*_ = *v*_*com*_ = 56.7 cm/s. When $c_{s}^{\left(+\right)}$ < $c_{s}^{\left(-\right)}$, the mean position of the vehicle body raises, *v*_*ext*_ > *v*_*com*_. It can also be seen from Table 1 that the larger the vehicle body’s shifting height is, the greater the difference in the vehicle body’s maximum speeds between the compression travel and extension travel are. For example, when the vehicle body is dropped by 4, 3, 2 and 1 cm, respectively, the differences between *v*_*com*_ and *v*_*ext*_ are 9, 4.1, 2.5 and 1.5 cm/s, respectively.

**Table 1:** The maximum velocities of the vehicle body in the extension travel and compression travel.

$c_{s}^{\left(+\right)}$ (N⋅s/m)	$c_{s}^{\left(-\right)}$ (N⋅s/m)	*v*_*ext*_ (cm/s)	*v*_*com*_ (cm/s)	Changes in mean position (cm)
6000	2000	39.9	48.9	-4
6000	3000	43.9	48.0	-3
6000	4000	48.2	50.7	-2
6000	5000	52.5	54.0	-1
6000	6000	56.7	56.7	0
2000	6000	47.2	39.9	4
3000	6000	49.9	44.0	3
4000	6000	52.2	48.2	2
5000	6000	54.1	52.5	1

Therefore, the explanation for the changes in the mean position of the vehicle body caused by damping asymmetric characteristic from the perspective of energy conservation is: when the damping coefficient in the extension travel is greater than that in the compression travel, the damper consumes more energy in the extension travel, causing the elastic potential energy stored in the suspension spring in the compression travel to be consumed more in the extension travel, at the same time, the maximum velocity of the vehicle body in the extension travel is reduced under the high damping state, the vehicle body cannot reach the highest point of motion in the previous cycle, resulting in the downward shift in the mean position of the vehicle body. Similarly, when the damping coefficient in the extension travel is smaller than that in the compression travel, the damper consumes more energy in the compression travel, and the potential energy stored by the suspension spring in the extension travel is consumed more in the compression extension, at the same time, the maximum velocity of the vehicle body in the compression travel is reduced, and the vehicle body cannot reach the lowest point of motion in the previous cycle, resulting in an upward shift in the mean position of the vehicle body.

### 3.2. Solution under random road excitation

The filtered white noise method is adopted as the time-domain model of road input [27].

$$\dot{q}(t)=-2\pi f_{0}q(t)+2\pi \sqrt{G_{q}(n_{0})v}w(t)$$ (7)

where *q*(*t*) is the vertical displacement of the road; *v* is the vehicle driving speed; *G*_*q*_(*n*_0_) is the road roughness coefficient; the lower cut-off frequency *f*_0_ is 0.1; *w*(*t*) is Gaussian white noise with a mean value of 0 and variance of 1.

#### 3.2.1. Feasibility verification

**Fig 9:**
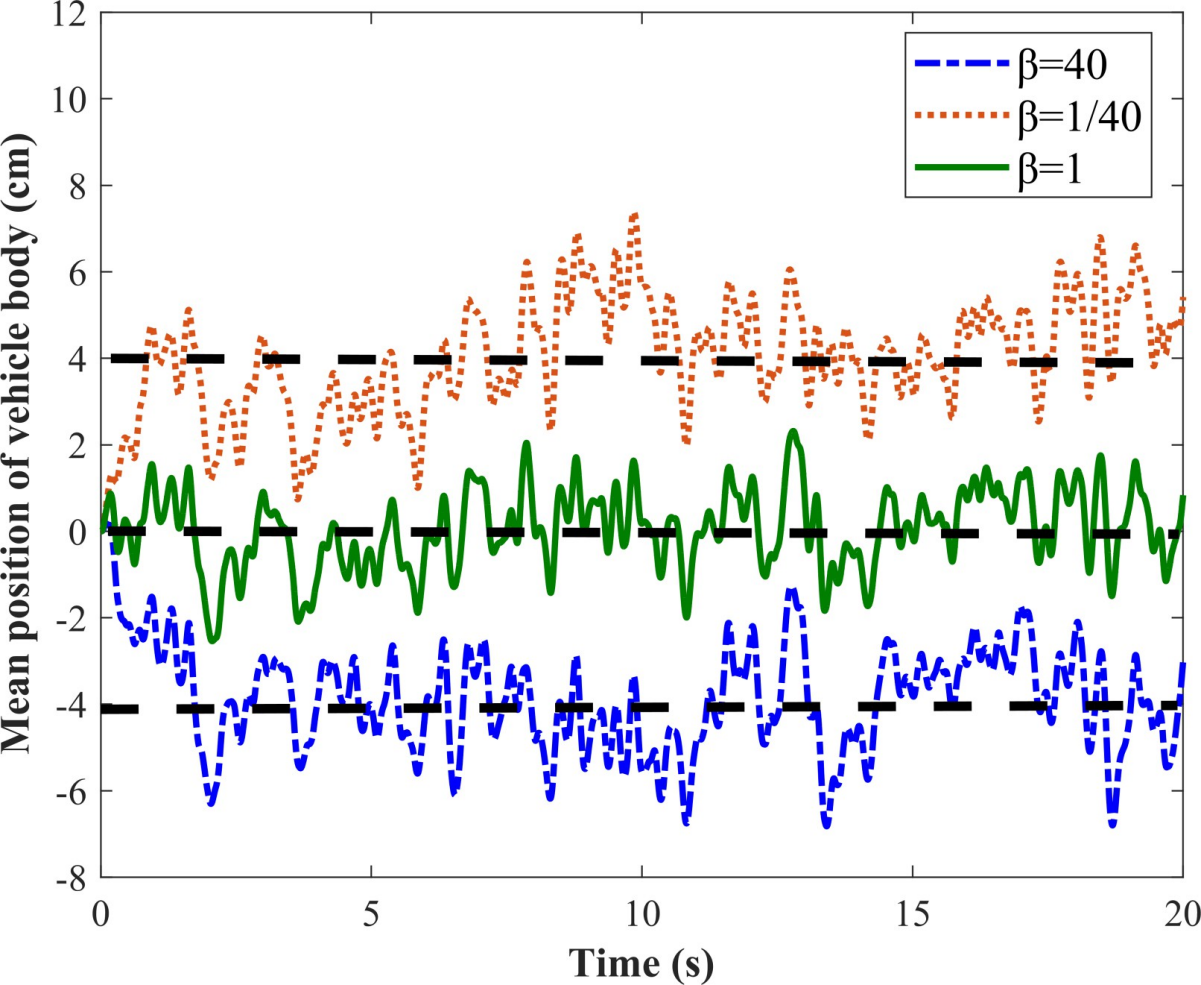
Vehicle body displacement responses under random road input.

The vehicle travels at a speed of 20 m/s on B-class road, *β* are taken as 40 and 1/4 respectively. As shown in Fig 9, when *β* = 40, the mean position of the vehicle body drops by 4 cm. When *β* = 1/40, the mean position of the vehicle body raises by 4 cm. It can be seen that the proposed method for shifting the vehicle body by utilizing the damping asymmetric characteristic is feasible under the random road excitation.

#### 3.2.2. Influence laws of parameters

**Fig 10:**
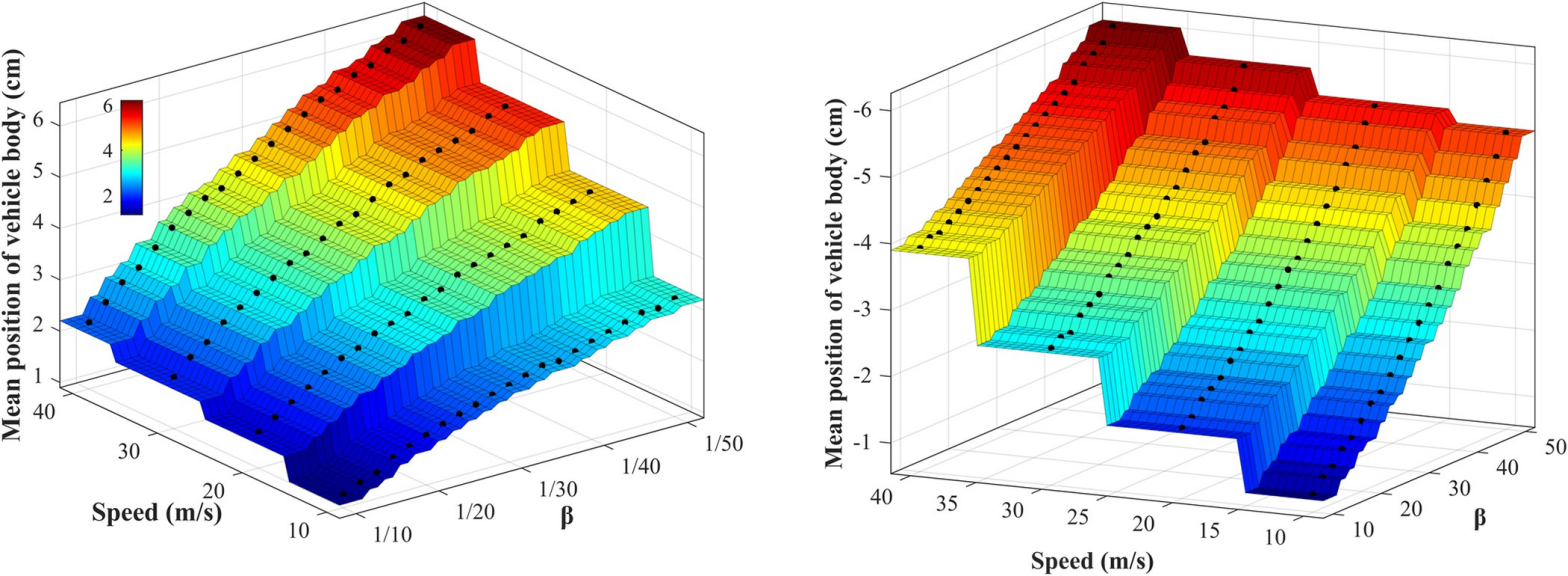
Vehicle body’s mean position with different speeds and *β*. (A) Vehicle body raises; (B) Vehicle body drops.

The vehicle is running on B-class road at speeds of 10, 20, 30, and 40 m/s, respectively. During the vehicle body raising adjustment, set $c_{s}^{\left(+\right)}$ = 50000 *β* N⋅s/m, $c_{s}^{\left(-\right)}$ = 50000 N⋅s/m, so the range of *β* is (0 1]. As shown in Fig 10A, the raising height of the vehicle body increases with the increase of vehicle speed and decreases with the increase of *β*. During the vehicle body dropping adjustment, set $c_{s}^{\left(+\right)}$ = 1000 *β* N⋅s/m, $c_{s}^{\left(-\right)}$ = 1000 N⋅s/m, so the range of *β* is [1 50]. As shown in Fig 10B, the vehicle body’s dropping height increases with the increase of vehicle speed and *β*.

**Fig 11:**
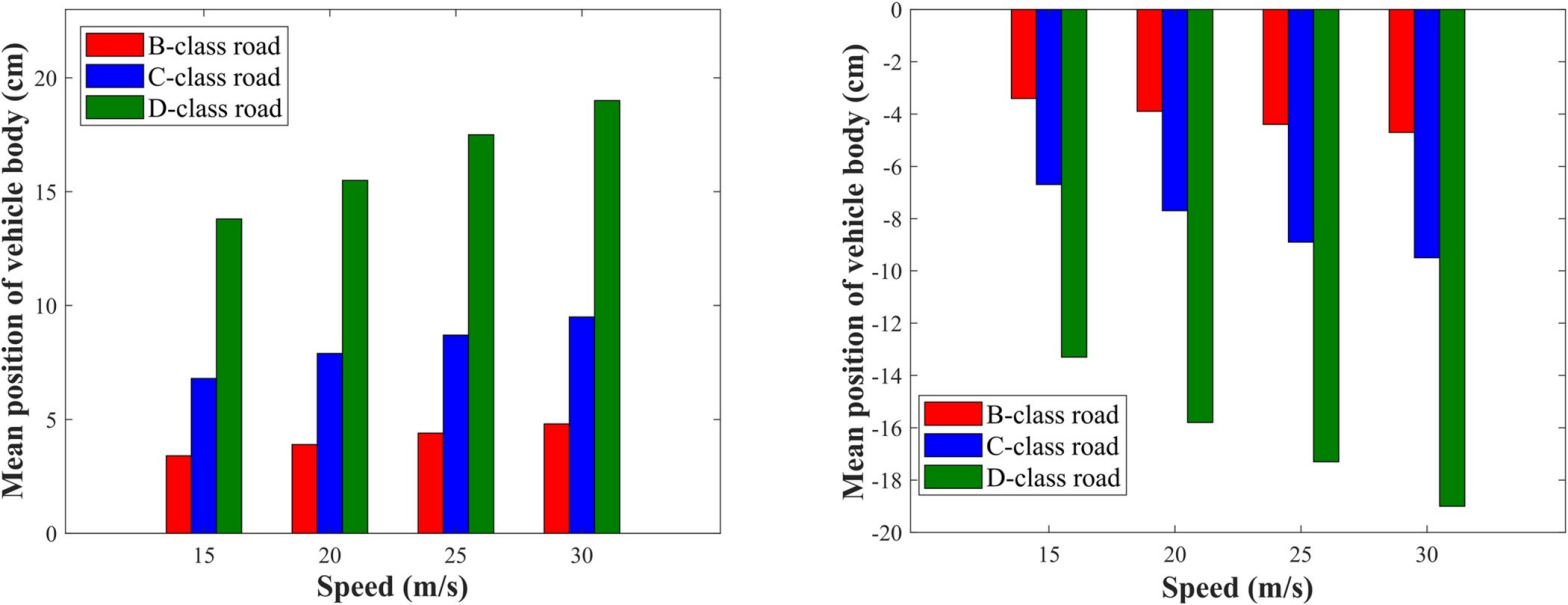
Vehicle body’s mean position with different speeds and road levels. (A) Vehicle body raises; (B) Vehicle body drops.

B, C, and D-class roads are adopted, respectively, and the speeds are 15, 20, 25, and 30 m/s. During the vehicle body raising adjustment, take *β* = 1/40; and during the vehicle body dropping adjustment, take *β* = 40. The raising and dropping heights of the vehicle body are shown in Fig 11A and 11B, respectively. It can be seen from the figures that when *β* is certain. At the same speed, the higher the road level is, the greater the vehicle body’s shifting height is. At the same road level, the higher the speed is, the greater the vehicle body’s shifting height is.

**Fig 12:**
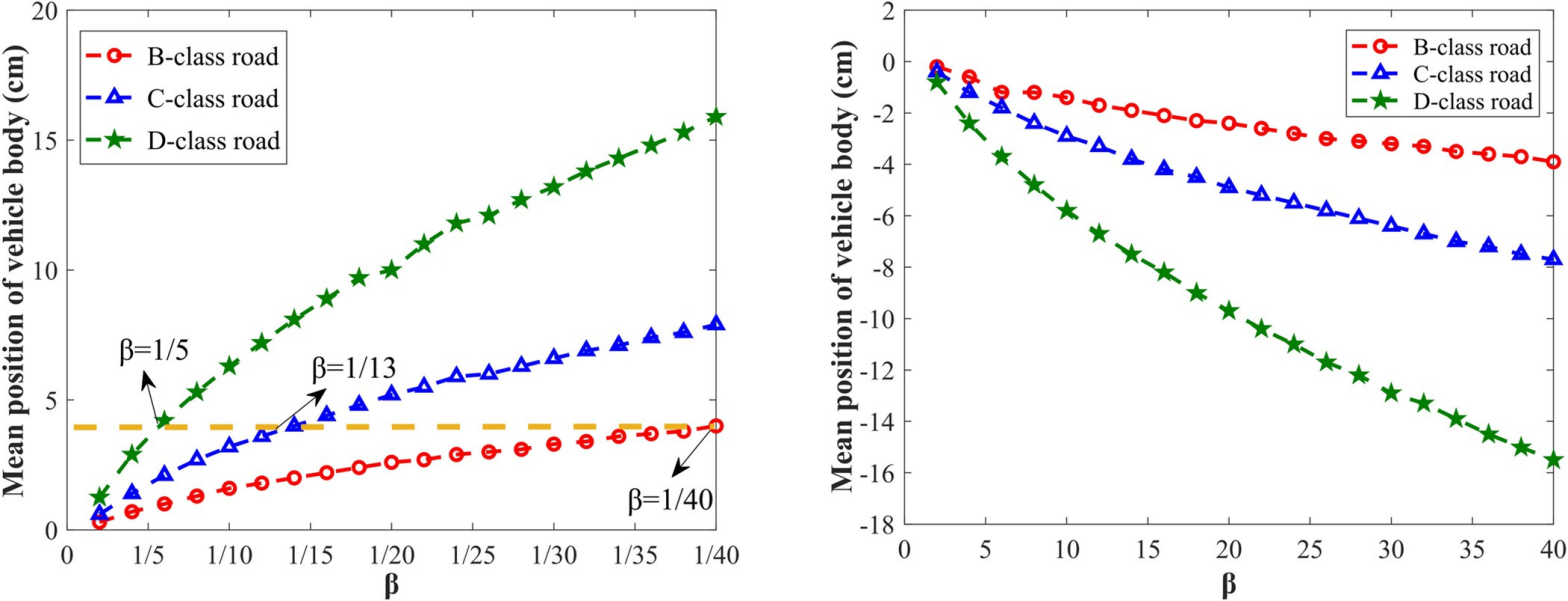
Vehicle body’s mean position with different *β* and roads. (A) Vehicle body raises; (B) Vehicle body drops.

The proposed method adopts a damping-adjustable damper to adjust the damping asymmetry ratio *β* to control the vehicle body’s shifting height, so the effects of *β* on the vehicle body’s shifting height need to be studied to further verify the feasibility of the method. Fig 12 shows the variation curves of the vehicle body’s shifting height with *β* when the vehicle travels on B, C, and D class roads at the speed of 20 m/s. Under the same road level, the raising height of the vehicle body increases with the decrease of *β*, while the vehicle body’s dropping height increases with the increase of *β*. When *β* is certain, the more roughness road is, the larger the vehicle body’s shifting height is. In practical applications, different *β* can be selected for different road inputs to achieve the expected vehicle body height. For example, as shown in Fig 12A, for B-class and C-class roads, to raise the vehicle body by 4 cm, the required damping asymmetric ratios *β* are 1/40 and 1/13, respectively. But for D-class road, to raise the vehicle by 4 cm, the required *β* is only 1/5.

In summary, under the random road input, the vehicle body’s shifting height increases with the increase of the vehicle speed and the road level, and the vehicle body height can be adjusted to the expected height by controlling the damping asymmetric ratio *β*. When the roughness of the road is relatively lower, a large damping coefficient is required to adjust the vehicle body to the expected height. For example, when the vehicle travels on a B-class road at a speed of 10 m/s, the required damping coefficient in the compression travel $c^{\left(-\right)}$ for raising the vehicle body by 4 cm is 40000 N⋅s/m. In practical applications, magnetorheological dampers (MRD) can be selected as damping-adjustable dampers, where the damping coefficient is controlled by changing the input current. The MRD based on shear mode and hybrid mode can reach a large damping coefficient, which can satisfy the damping coefficient required in this paper to adjust the vehicle body height [28, 29].

## 4. Vehicle height control based on third-order LADRC

Common control methods include optimal control [30], sliding mode control [31], PID control [32], and fuzzy control [33], etc. Gao [34] used the concept of frequency scale to linearize the traditional active disturbance rejection control and proposed linear active disturbance rejection control (LADRC). LADRC combines the parameter tuning of the controller with the bandwidth and estimates the external disturbance by increasing the expansion state of the observer, and actively estimating, offsetting, and compensating to achieve the active disturbance rejection performance of the system. LADRC does not rely on the accurate mathematical model and has strong applicability and robustness, which has been widely used [35–37]. In this paper, a third-order LADRC is designed to realize vehicle height control.

### 4.1. Control strategy

The 2-DOF vehicle model shown in Fig 3 can be written as

$$\left\{ \begin{array}{l} m_{s}{\ddot{z}}_{s}+k_{s}(z_{s}-z_{u})+c_{c}({\dot{z}}_{s}-{\dot{z}}_{u})+f_{d}^{}=0\\ m_{u}{\ddot{z}}_{u}+k_{s}(z_{u}-z_{s})+k_{u}(z_{u}-z_{r})+c_{c}({\dot{z}}_{u}-{\dot{z}}_{s})-f_{d}^{}=0 \end{array}\right.$$ (8)

Based on the proposed method for shifting the vehicle body by utilizing the damping asymmetric characteristic, the vehicle body’s raising and dropping switch functions are designed, respectively.

The vehicle body’s raising switch function

$$f_{d}=\{\begin{array}{l} c_{large}({\dot{z}}_{s}-{\dot{z}}_{u})\;{\dot{z}}_{s}-{\dot{z}}_{u}>0\\ c_{small}({\dot{z}}_{s}-{\dot{z}}_{u})\;{\dot{z}}_{s}-{\dot{z}}_{u}\leq 0 \end{array}$$ (9)

The vehicle body’s dropping switch function

$$f_{d}=\{\begin{array}{l} c_{small}({\dot{z}}_{s}-{\dot{z}}_{u})\;{\dot{z}}_{s}-{\dot{z}}_{u}>0\\ c_{l\arg e}({\dot{z}}_{s}-{\dot{z}}_{u})\;{\dot{z}}_{s}-{\dot{z}}_{u}\leq 0 \end{array}$$ (10)

where *c*_*small*_ is the small damping coefficient, *c*_*l*arg*e*_ is the large damping coefficient.

**Fig 13:**
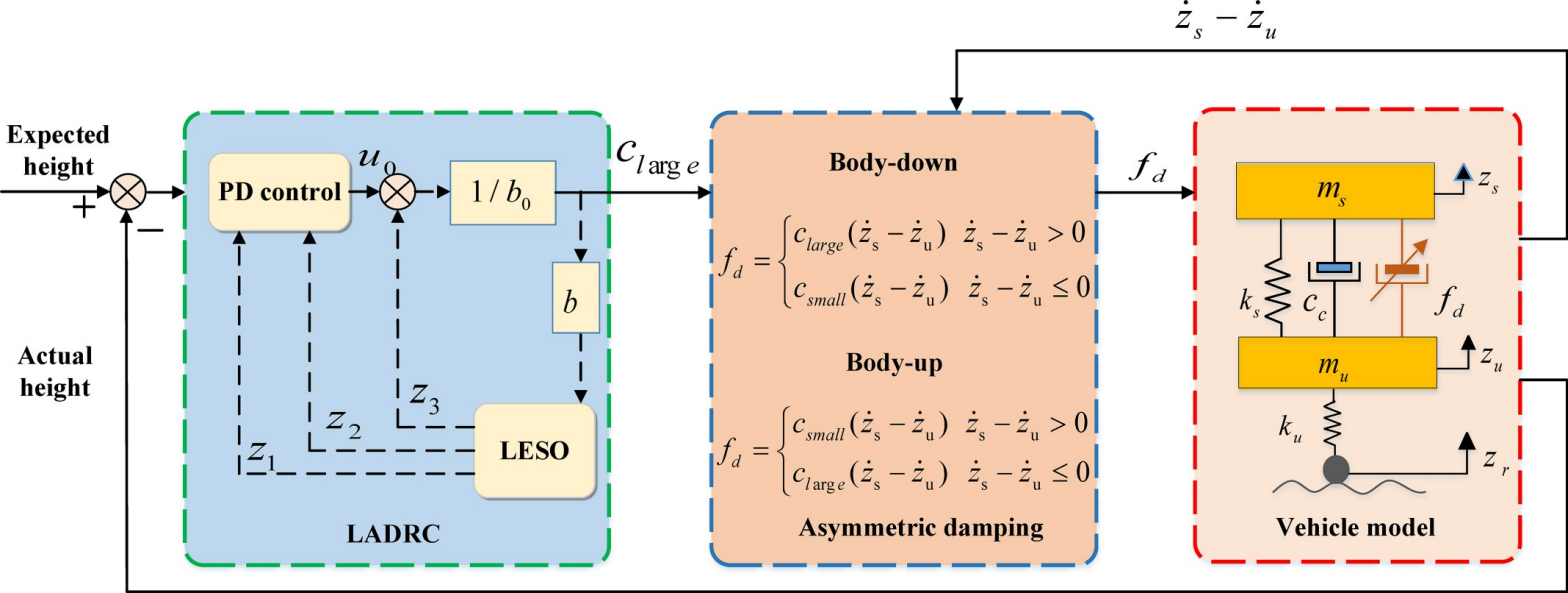
Schematic diagram of the vehicle height control.

Fig 13 shows a schematic diagram of the vehicle shifting control based on the third-order LADRC, the expected vehicle height is determined according to different road conditions and vehicle speeds, and the deviation between the expected vehicle height and the actual vehicle height is used as the input of LADRC, fix the small damping coefficient *c*_*small*_ = 1000 N⋅s/m, *c*_*l*arg*e*_ is obtained by LADRC, and the above vehicle body shifting switch functions are used for vehicle shifting control.

### 4.2. Design of LADRC

The second-order system represented by Eq (8) can be expressed as

$$\ddot{y}=-a\dot{y}-by+w+bu$$ (11)

where *a* and *b* are system parameters; *y* and *u* are output and input signals, respectively; *w* is an external disturbance.

Rewrite Eq (11) as

$$\ddot{y}=-a_{}\dot{y}-by+\omega +(b‐b_{0})u+b_{0}u=f(\dot{y},y,w)+b_{0}u$$ (12)

where *b*_0_ is the estimate of *b*, $f(y,\dot{y},\omega ,t)$ is the total disturbance of the system.

Set *x*_1_ = *y*, $x_{2}=\dot{y}$, $x_{3}=f(y,\dot{y},w,t)$, the system state equation can be written

$$\left\{ \begin{array}{l} {\dot{x}}_{1}=x_{2}\\ {\dot{x}}_{2}=x_{3}+b_{0}u\\ {\dot{x}}_{3}=\dot{f}(y,\dot{y},w,t) \end{array}\right.$$ (13)

where *x*_1_ and *x*_2_ are the state variables of the system, *x*_3_ is the expansion state of the system.

The linear extended state observer (LESO) is established as follows

$$\left\{ \begin{array}{l} e=y-z_{1}\\ {\dot{z}}_{1}=z_{2}+\beta_{1}e\\ {\dot{z}}_{2}=z_{3}+\beta_{1}e+b_{0}u\\ {\dot{z}}_{3}=\beta_{3}e\\ \hat{y}=z_{1} \end{array}\right.$$ (14)

where *z*_1_, *z*_2_ and *z*_3_ are observation values of the three state variables *x*_1_, *x*_2_ and *x*_3_; $\hat{y}$ is the observation values of system output *y*, by selecting the appropriate observer gains *β*_1_, *β*_2_ and *β*_3_, the LESO can realize the real-time tracking of the variables in the Eq (12), that is, *z*_1_→*y*, $z_{2}\to \dot{y}$, $z_{3}\to f(y,\dot{y},w)$.

The characteristic polynomial of LESO is

$$\lambda (s)=s^{3}+\beta_{1}s^{2}+\beta_{2}s+\beta_{3}={\left(s+w_{0}\right)}^{3}$$ (15)

*β*_1_ = 3*ω*_*o*_, $\beta_{2}=3\omega_{o}^{2}$ and $\beta_{3}=\omega_{o}^{3}$ are selected so that the characteristic polynomial (15) is Hurwitz stable [38]. where *ω*_*o*_ is the observer bandwidth.

The output of LADRC *c*_*l*arg*e*_ is expressed as

$$c_{l\arg e}=\frac{-z_{3}+u_{0}}{b_{0}}$$ (16)

Neglecting the estimation error of *z*_3_ to $f(y,\dot{y},w)$, Eq (12) can be simplified to a series integral structure

$$\ddot{y}=f(y,\dot{y},w)-z_{3}+u_{0}\approx u_{0}$$ (17)

Feedback control rate *u*_0_ adopts linear PD combination form

$$u_{0}=k_{p}(r_{e}-z_{1})-k_{d}z_{2}$$ (18)

where *r*_*e*_ is the expected vehicle height; *k*_p_ and *k*_d_ are the proportional gains of the controller.

Substituting Eq (18) Eq (17), the closed-loop transfer function of the system can be obtained

$$\psi (s)=\frac{k_{p}}{s^{2}+k_{d}s+k_{p}}$$ (19)

Select *k*_p_ = $w_{c}^{2}$, *k*_d_ = $2w_{c}^{2}$ [39], the system can be guaranteed to be bounded input-output stable by selecting the appropriate controller bandwidth *w*_*c*_.

### 4.3. Simulation results

To verify the effectiveness of the established LADRC-based vehicle height control system, simulation verification is carried out under the sinusoidal and random roads, respectively, and the vehicle parameters are selected: *m*_*s*_ = 410 kg, *m*_*u*_ = 39 kg, *k*_*s*_ = 20000, *k*_*u*_ = 183000 N/m, *c*_*c*_ = 1200 N⋅s/m [25]. According to the vehicle’s height, three modes of ‘low’, ‘median’, and ‘high’ are set, corresponding to the coordinates of -5 cm, 0 cm, and 5 cm, respectively.

#### Condition 1: Sinusoidal road

**Fig 14:**
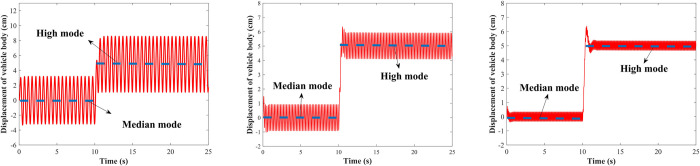
Median mode to high mode under the sinusoidal road. (A) *f* = 2 Hz; (B) *f* = 5 Hz; (C) *f* = 10 Hz.

**Fig 15:**
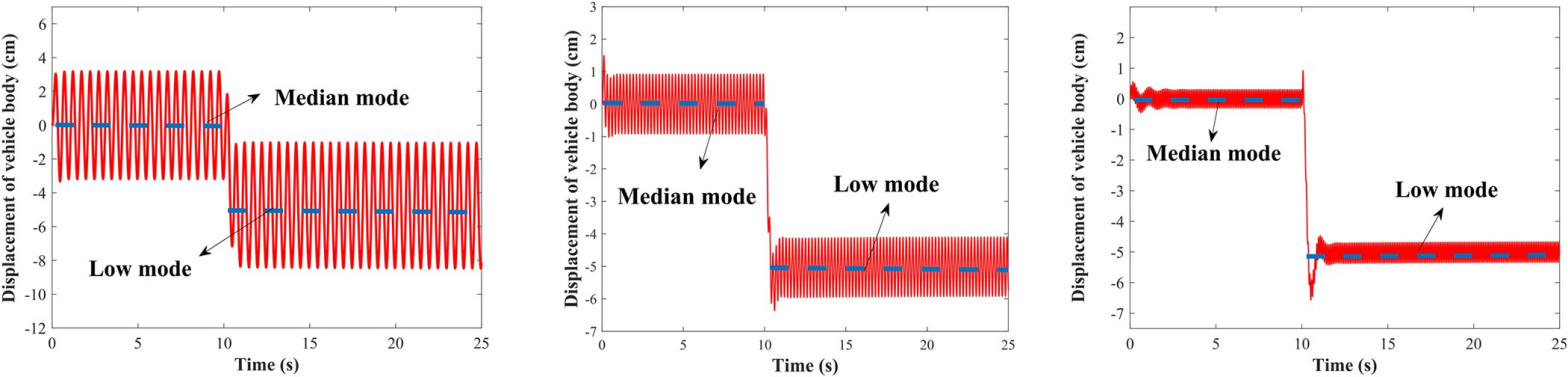
Median mode to low mode under the sinusoidal road. (A) *f* = 2 Hz; (B) *f* = 5 Hz; (C) *f* = 10 Hz.

The road excitation is performed with a sine wave of the amplitude of 3 cm and frequencies of 2 Hz, 5 Hz, and 10 Hz, respectively. Fig 14 shows the vehicle body regulation results from the median to the high mode with a regulation time of 25 s. The initial position is in the median mode, and the vehicle body is controlled to raise at 10 s, the vehicle can reach the high mode from the median mode within 1 s, affected by the road excitation, it oscillates up and down at the target height and remains stable. Fig 15 shows the regulation from median to low mode. It can be seen that the LADRC-based vehicle height adjustment system can quickly and accurately adjust the vehicle body to the target height under the sinusoidal roads.

**Fig 16:**
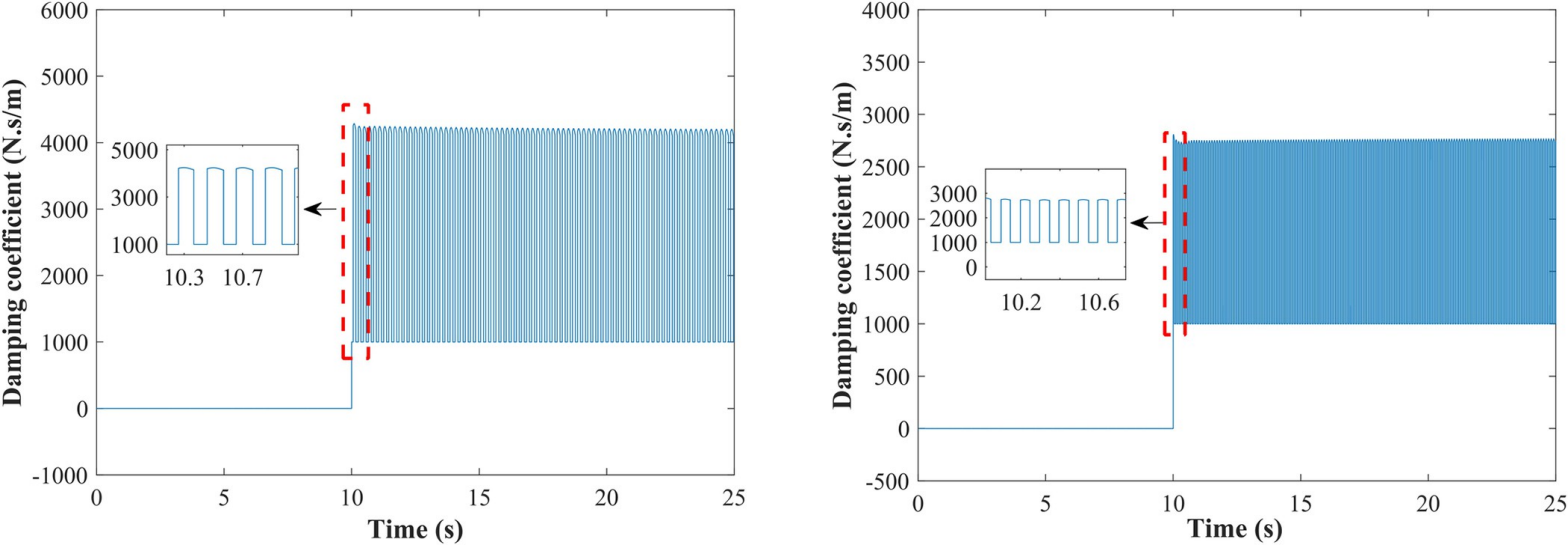
Damping coefficient of the damping-adjustable damper from median to high mode. (A) *f* = 5 Hz.; (B) *f* = 10 Hz.

Using a sine wave with an amplitude of 3 cm and frequencies of 5 Hz and 10 Hz as the road excitation, the damping coefficients of the adjustable-damping damper when the vehicle body is raised from the median mode to the high mode are shown in Fig 16. As can be seen from the figure, when the road excitation frequency is 5 Hz, the maximum damping coefficient is 4000 N⋅s/m, and the minimum damping coefficient is 1000 N⋅s/m, the damping coefficient switches rapidly between large and small damping coefficients; when the road excitation frequency is 10 Hz, the maximum damping coefficient and the minimum damping coefficient are 2800 N⋅s/m and 1000 N⋅s/m, respectively. The general damping-adjustable damper can achieve the maximum damping coefficient required for control.

**Fig 17:**
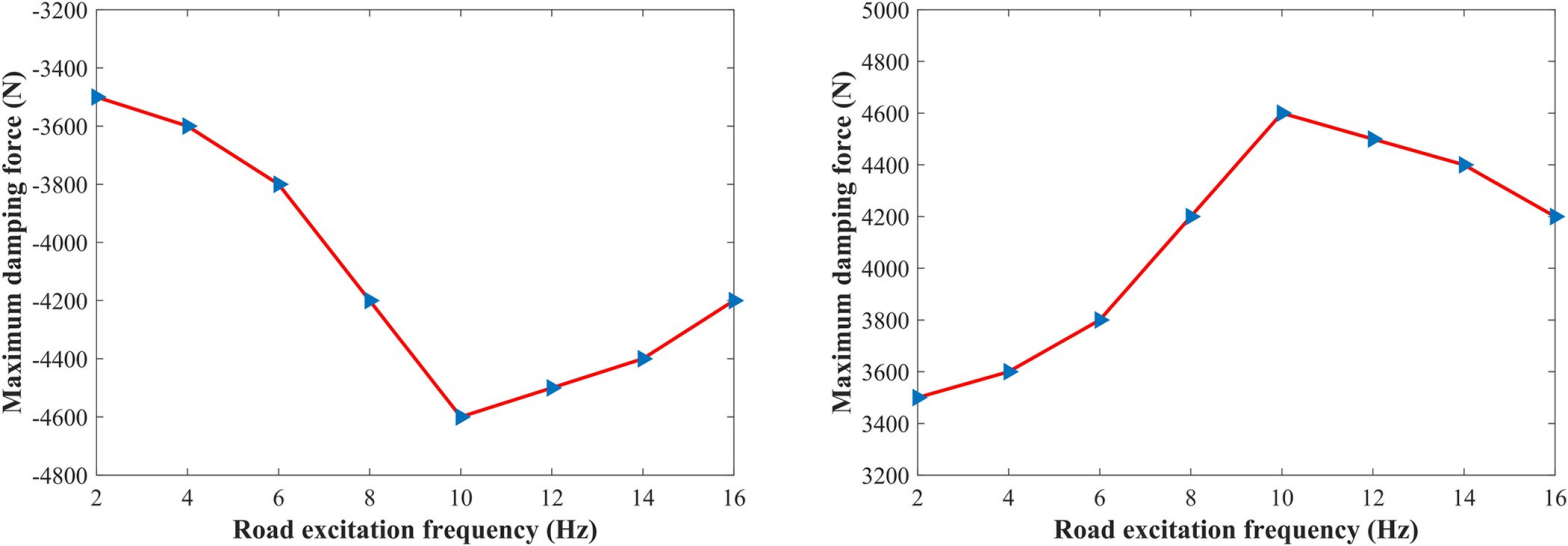
Maximum damping force required for shifting the vehicle body at different road excitation frequencies. (A) Vehicle body raises; (B) Vehicle body drops.

To comprehensively test the feasibility of the proposed method for shifting the vehicle body, the maximum damping forces which are required for shifting the vehicle body by 5 cm under different road excitation frequencies are simulated. Fig 17A and 17B show the maximum damping force required for raising and dropping the vehicle body by 5 cm, respectively, the damping force in the extension travel is positive, and the damping force in the compression travel is negative, as can be seen from the figures, with the increase of the excitation frequency, the absolute value of the maximum damping force first increases and then decreases, reaching a maximum value near 10 Hz, after calculation, the wheel’s inherent frequency is 11 Hz, the required maximum damping force reaches the maximum near the wheel’s inherent frequency, which is 4600 N, the damping force of the general damping-adjustable damper can reach the damping force which is required for controlling the vehicle shifting height.

#### Condition 2: Random road

**Fig 18:**
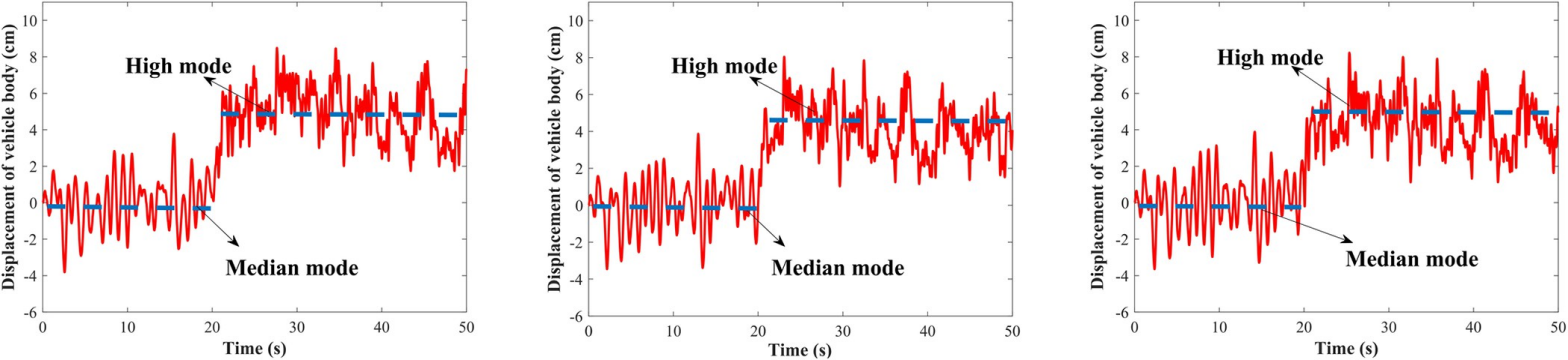
Median mode to high mode under the random road. (A) 20 m/s; (B) 30 m/s; (C) 40 m/s.

**Fig 19:**
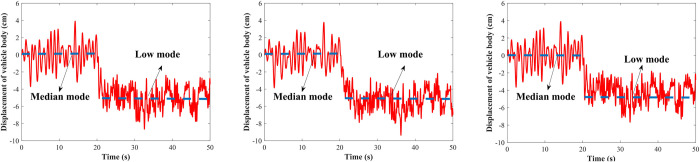
Median mode to low mode under the random road. (A) 20 m/s; (B) 30 m/s; (C) 40 m/s.

The vehicle is set to drive at speeds of 20, 30, and 40 m/s on a B-class road, and the road input time-domain model represented by Eq (7) is used. Figs 18 and 19 show the vehicle displacement from the median to the high mode and from the median to the low mode with a simulation time of 50 s, respectively. The initial position is in the median mode (0 cm), the shifting control is performed on vehicle at 20 s, and the vehicle is quickly shifted to the target height within 1 s. It can be seen that the proposed vehicle body shifting methods can shift the vehicle body to the target height quickly under the random roads.

**Fig 20:**
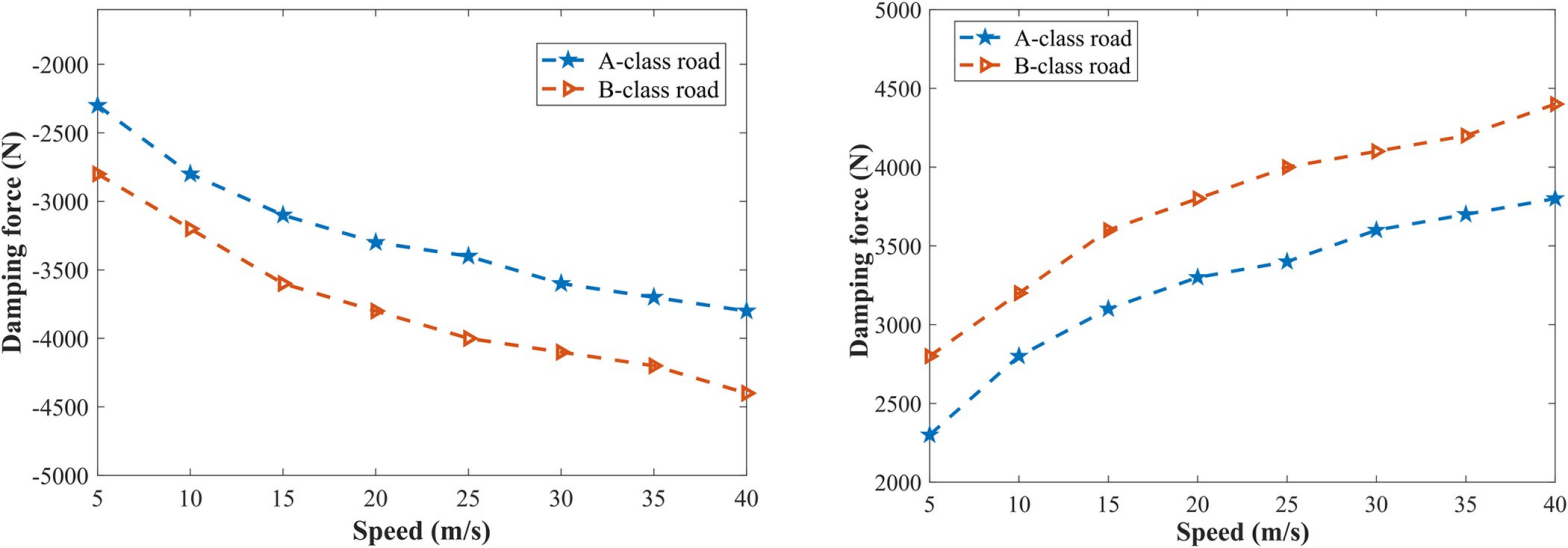
Maximum damping forces required for shifting the vehicle body by 5 cm. (A) Vehicle body raises; (B) Vehicle body drops.

To verify the feasibility of vehicle height adjustment system based on LADRC in practical application, Fig 20 shows the maximum damping forces which are required for shifting the vehicle body by 5 cm at different speeds on A and B-class roads. It can be seen that the absolute value of the required maximum damping force increases with the increase of speed under the same road level, and the required maximum damping force on B-class is greater than that on A-class road at the same vehicle speed. When the vehicle is traveling on a B-class road at speed of 40 m/s, the required maximum damping force reaches a maximum of 4400 N, a general damping-adjustable damper can achieve the damping force required for controlling the vehicle body height. In addition, the vehicle body’s weight of the studied vehicle model is large, the heavier the vehicle body is, the greater the damping force required for shifting the vehicle is, for light vehicles, the damping force that is required for shifting the vehicle body by 5 cm will be significantly reduced.

## 5. Conclusions

1. This paper proposes a new method for shifting the vehicle body by utilizing the damping asymmetric characteristic. A single-wheel vehicle model is established, and the system’s dynamic response are obtained by the fourth-order Runge-Kutta method. The feasibility of the proposed method is preliminarily verified, and the influences of key parameters on the vehicle body’s shifting height are explored. The results show that under sinusoidal road input, the vehicle body’s shifting height increases with the increase of excitation amplitude, increases and then decreases with the increase of excitation frequency, reaching the maximum near the wheel’s inherent frequency. At the random road input, the vehicle body’s shifting height increases with the increase of the vehicle speed and road level. The vehicle body height can be controlled to the expected height by adjusting the damping asymmetric ratio at both sinusoidal and random roads.
2. The internal mechanism of the change in the mean of the vehicle body caused by damping asymmetric characteristic is revealed from the perspective of energy conservation: the large damping will cause the damper to consume more energy, resulting in more dissipation of the elastic potential energy of the suspension spring. At the same time, the maximum velocity of the vehicle body decreases under the condition of large damping, the vehicle body can not reach the highest (low) point in the previous cycle, thus changing the mean position of the vehicle body vibration.
3. Based on the basic idea of shifting the vehicle height by utilizing the damping asymmetric characteristic, a third-order LADRC is designed. The simulation results show that the LADRC-based vehicle body shifting method can quickly and accurately shift the vehicle body to the expected height and maintain stability under sinusoidal road and random road input, realizing the purpose of using damped-adjustable dampers to control the vehicle height without installing additional vehicle shifting devices. The proposed vehicle body shifting method tries a new way for the study of the vehicle height control.

Future work will build a vehicle vibration test bench to further verify the feasibility and validity of the proposed method for controlling the vehicle height by utilizing the damping asymmetric adjustment via the semi-active suspension.

## Supporting information

S1 File (ZIP)
